# Supplementary material for: UV-Excited Luminescence in Porous Organosilica Films with Various Organic Components
Source: Nanomaterials (Basel). 2023 Apr 20;13(8):1419. doi: 10.3390/nano13081419 (PMC10143820; doi:10.3390/nano13081419)
Supplement: Supplementary file 1 [file nanomaterials-13-01419-s001.zip › nanomaterials-2322129-supplementary.pdf]

nanomaterials

MDPI

Supplementary Materials

Table S1. Physical properties of organosilicate glass (OSG) films: first type: both ethylene bridges and methyl terminal groups with different precursor ratios (BTMSE/MTMS = 47/53 and 25/75); second type: a mixture of 1,3,5- and 1,3-benzene groups with a ratio of 1,3,5/1,3 = 1:3, 1,3,5/1,3 = 1:7 and 1,4-benzene bridges without porogen (1,4-BB) and with 30 wt% porogen (1,4-BB-p); third type: contains only methyl terminals without bridging groups (MTMS – without porogen, MTMS-p – with porogen). All films are completely cured in the air.

OSG films	EP data						FTIR data
	Porogen concentration (wt%)	*d* (nm)	*RI*	Open porosity (%)	Pore radius (nm)	Surface area (m²/cm³)	Si–CH3/ Si–O–Si ratio
BTMSE/MTMS = 47/53	0	478.0	1.397	1.2	0.81	31.72	0.151
	10	493.6	1.349	12.2	0.81	303.26	0.142
	20	485.2	1.335	25.1	0.81	621.81	0.150
	30	465.0	1.306	32.5	1.42	459.89	0.183
	50	434.2	1.223	45.5	2.34	391.19	0.186
BTMSE/MTMS = 25/53	0	617.3	1.453	8.7	0.81	216.43	0.299
	10	622.0	1.370	12.4	0.81	307.78	0.274
	20	598.3	1.357	19.4	0.97	401.66	0.283
	30	629.2	1.308	27.7	1.42	391.73	0.290
	50	585.1	1.200	50.6	4.65	219.34	0.298
1,3,5/1,3BB (1:3)	0	254.9	1.477	-	-	-	0.003
	17	356.4	1.316	28.9	0.81	716.39	0.026
	23	402.5	1.285	38.7	0.81	958.04	0.033
	29	420.1	1.270	42.4	0.81	1049.29	0.053
	33	416.3	1.268	42.9	0.92	935.01	0.059
	38	413.3	1.256	41.2	0.92	898.07	0.033
	41	398.9	1.285	37.9	1.1	691.59	0.053
1,3,5/1,3BB (1:7)	0	315.7	1.470	-	-	-	0.080
	17	377.7	1.324	26.2	0.81	649.56	0.014
	23	401.9	1.281	36.7	0.81	908.66	0.026
	29	405.4	1.272	40.4	0.81	999.99	0.058
	33	401.5	1.266	41.9	0.92	913.36	0.079
	38	419.3	1.259	41.9	0.92	913.25	0.038
	41	425.0		41.9	0.92	913.22	0.054
1,4-BB	0	177.0	1.25	10.3	0.92	229.56	0.001
			1.559				
1,4-BB-p	30	169.0	1.428	29.3	0.76	776.96	0.012
MTMS	0	266.0	1.363	7.5	0.71	215.02	0.048
MTMS-p	30	226.0	1.300	33.1	4.1	165.88	0.044

OSG – Organosilicate glass, EP – Ellipsometric Porosimetry, .
FTIR – Foriur Transform Infrared Spectroscopy, *d* – thickness, *RI* – Refractive index.

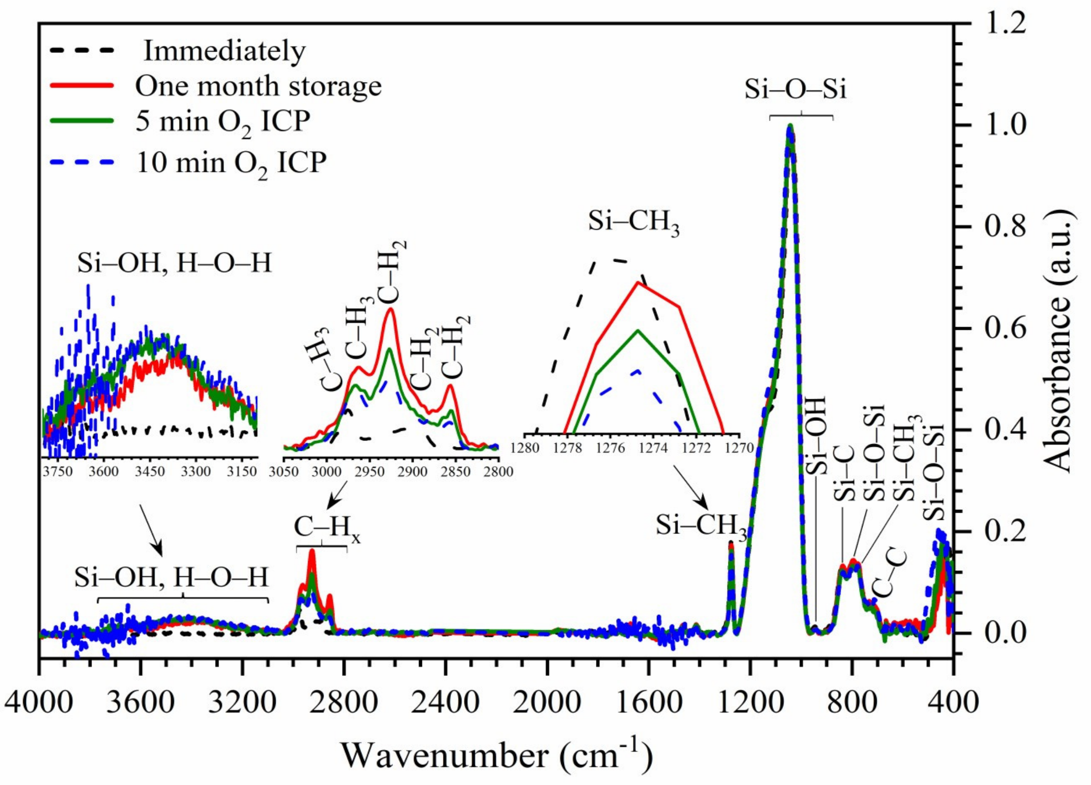

Figure S1. Effect of chemical composition during storage of film (BTMSE/MTMS = 47/53, .20 wt% Brij®30) in clean room environment and after soft oxygen ICP plasma. Loss of Si– CH_3 groups and hydrophilization was observed for films by using soft oxygen ICP plasma (remove the chemical residue). The thickness of low-k film before exposure to O_2 ICP plasma was equal to 466 nm , then it reduced to 435 nm after 5 min and 399 nm after 10 min. Meanwhile, the corresponding reductions of Si–CH_3/Si–O–Si peaks ratio were from 0.173 to 0.163 and to 0.154, respectively. The changes of hydrocarbon residues peaks in the region 2800-3000 cm^{-1} was about 3 times larger. These observation suggest that the used plasma conditions were sufficiently soft to minimize plasma damage of low-k matrix (small change Si–CH_3/Si–O–Si ratio) and the ICP generated oxygen radicals are mainly consumed on the accumulated carbon residue. The equivalent depth of CH_3 depleted region was estimated by using approach described in the paper (F. Leroy et al. Journal of Physics D Applied Physics 48(43):435202) and was equal to ≈27 nm after 5 min and ≈45 nm after 10 min plasma exposure.

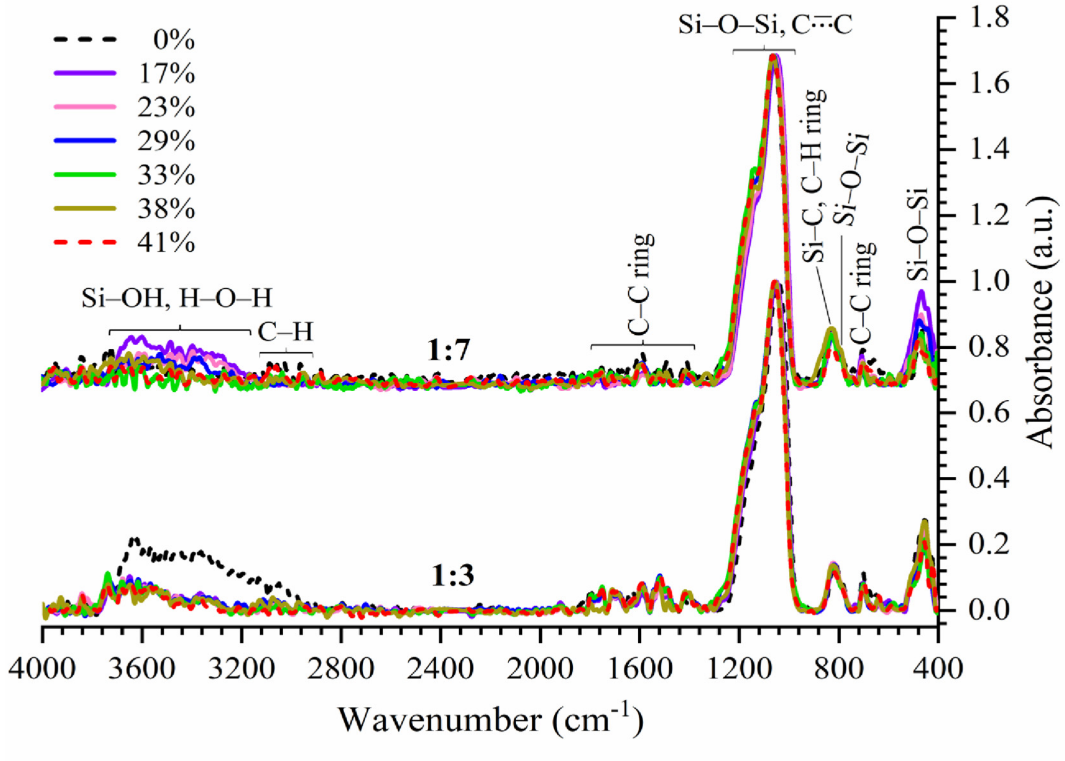

Figure S2. FTIR spectra of 1,3,5- and 1,3-benzene bridged ratio of 1:3 (bottom) and 1:7 (top) organosilica films with different porogen concentrations (0–41 wt%), soft bake at 200 °C and hard bake at 400 °C in air. The spectra are generally quite similar but the films containing less 1,3 benzene bridges (1:3) are more hydrophilic. .

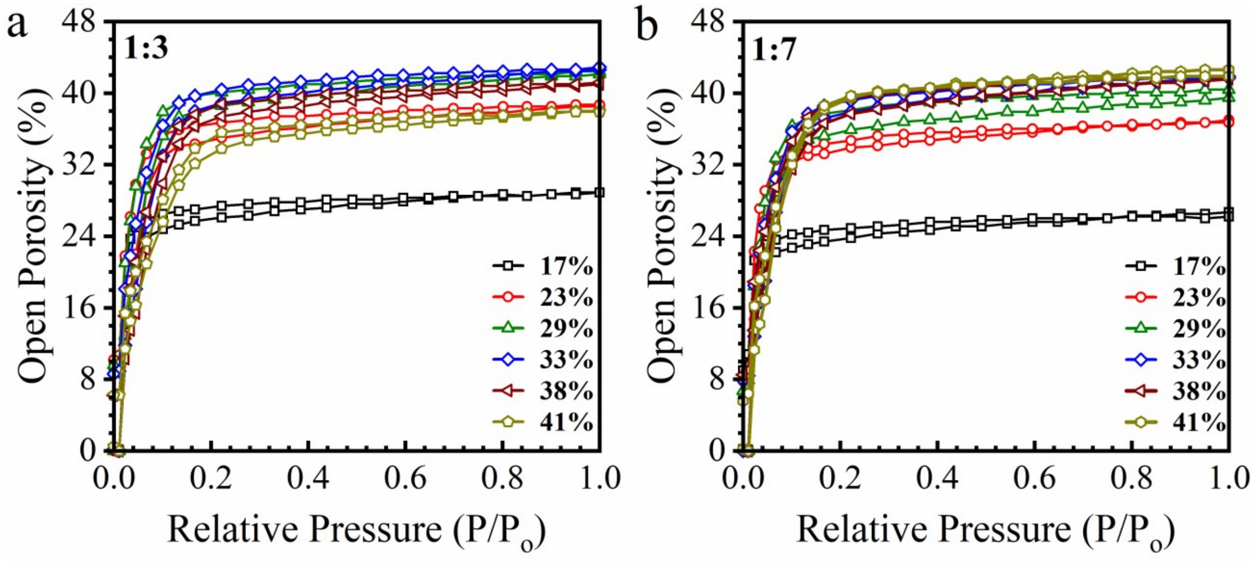

Figure S3. Adsorption-desorption isotherms for determining the value of open porosity, generated by ellipsometric porosimetry, of 1,3,5- and 1,3-benzene bridges ratio of 1:3 (a) and 1:7 (b) organosilica films with different porogen concentrations (17–41 wt%), soft bake at 150 °C and hard bake at 400 °C in air. The films containing less 1,3 benzene bridges (1:3) always have larger pore size at the same porogen concentration.

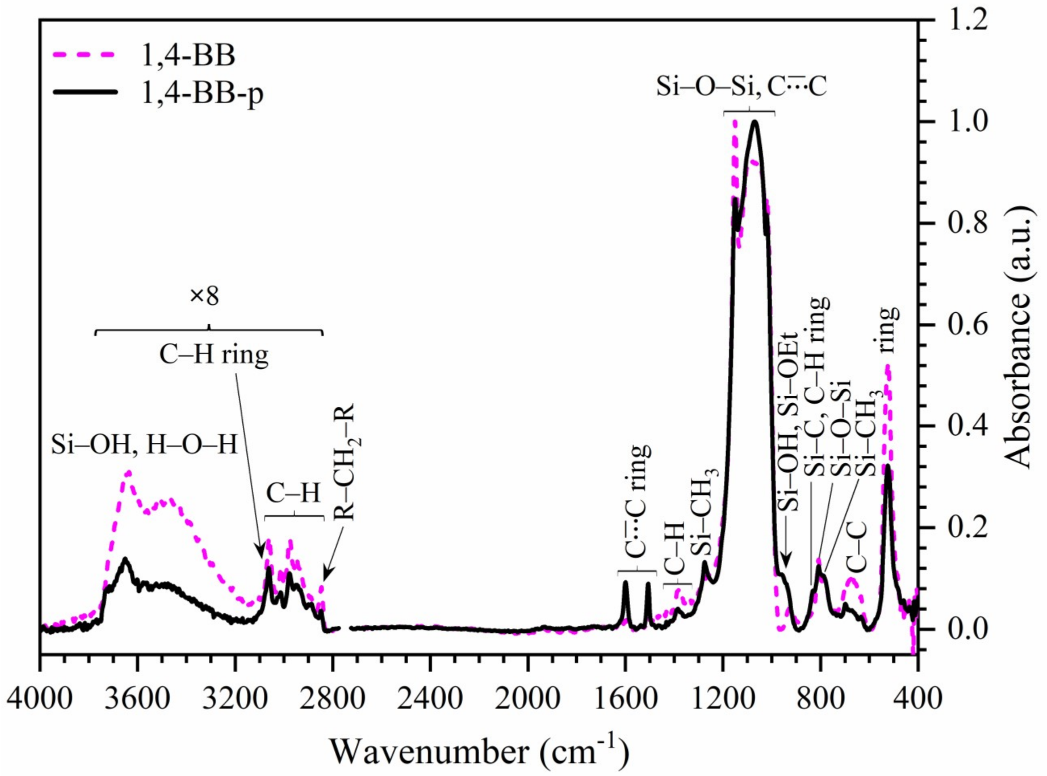

Figure S4. FTIR spectra of pure 1,4-benzene bridged organosilica films: dense (1,4-BB) and with 30 wt% porogen content 1,4-benzene bridged (1,4-BB-p) organosilica films, both the films were completely cured in air. The porous 1,4-BB-p film contains larger concentration of adsorbed water.

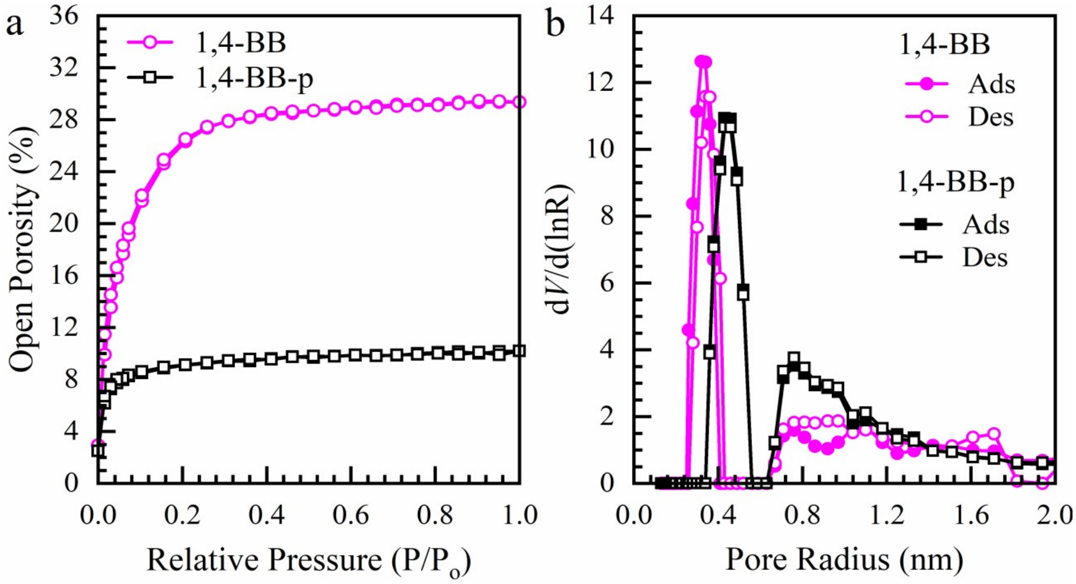

Figure S5. Adsorption-desorption isotherms for determining the value of open porosity (a) and pore size distribution (b), generated by ellipsometric porosimetry, of pure 1,4benzene bridged organosilica films: dense (1,4-BB) and with 30 wt% porogen content 1,4benzene bridged (1,4-BB-p) organosilica films, both the films were cured completely in air.

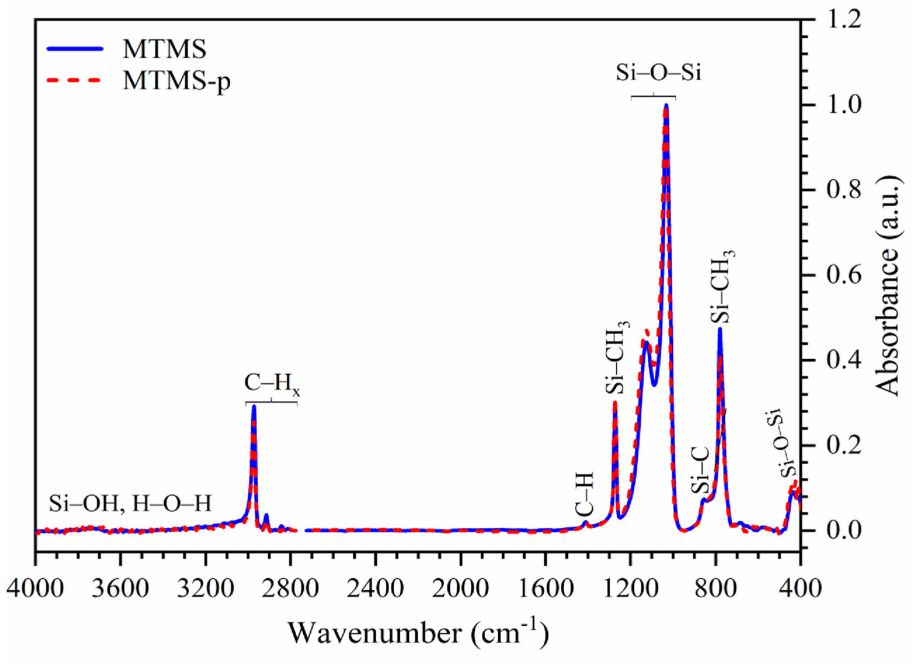

Figure S6. FTIR spectra of pure methyl-terminated organosilicate : dense (MTMS) and with 30 wt% porogen content methyl-terminated (MTMS-p), both the films were cured completely in air. The films with methyl terminal groups are much more hydrophobic than the films with benzene bridges, as expected.

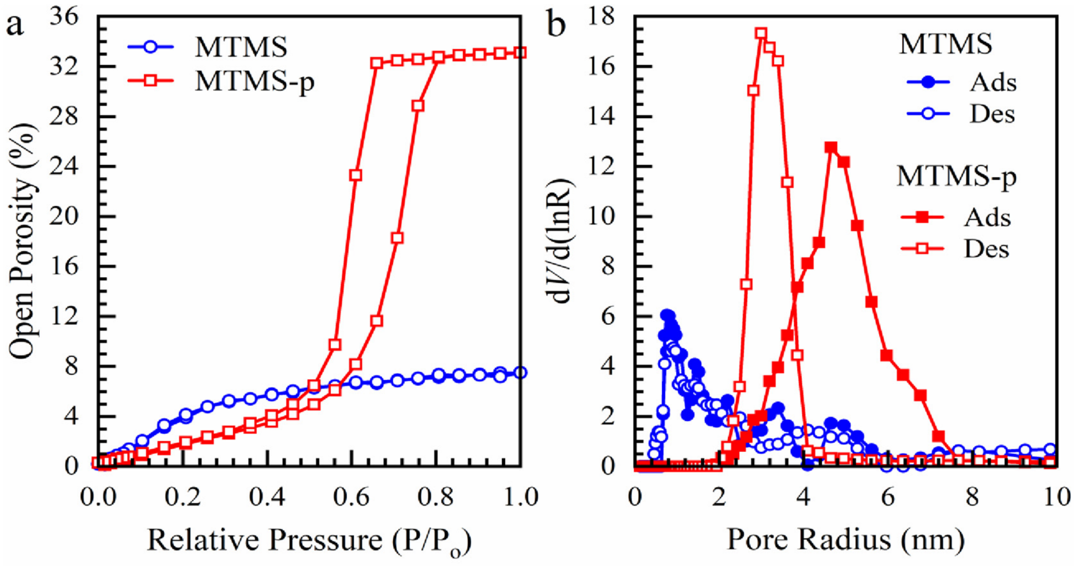

Figure S7. Adsorption-desorption isotherms for determining the value of open porosity (a) and pore size distribution (b), generated by ellipsometric porosimetry, of pure methylterminated organosilica films: dense (MTMS) and with 30 wt% porogen content methyl terminated (MTMS-p) organosilica films, both the films were cured completely in the air.

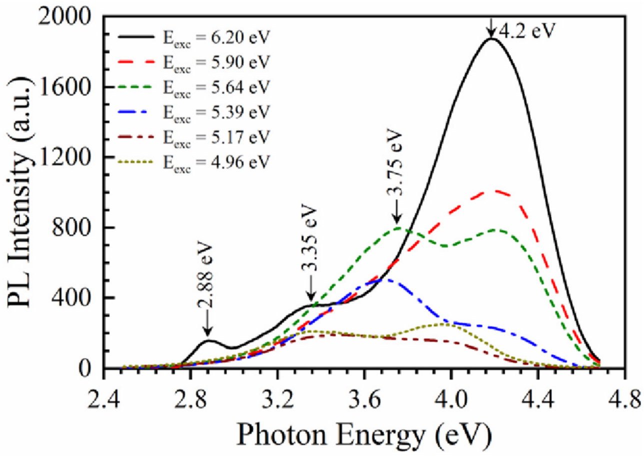

Figure S8. UV induced PL spectra measured at the different excitation energy for the ethylene bridged films (BTMSE/MTMS = 47/53).

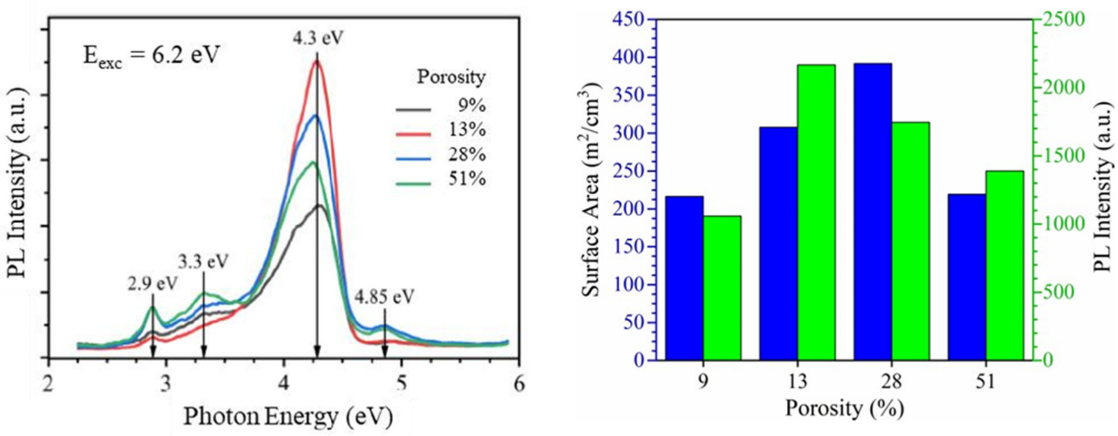

Figure S9. Correlation between 4.3 eV UV induced (upon excitation with light of 6.2 eV) room temperature PL emission with the measured surface area in hard baked OSG lowk films containing both methyl terminal and ethylene bridging groups (BTMSE/MTMS ratio of 25/75) with different porosity. However, the PL bands at 2.9, 3.3 and 4.85 eV have the highest intensity in highly porous films. It can be assumed that the reason is that the terminal methyl groups and hydrocarbon residues are mainly located on the pore wall surface. However, this assumption needs further study.
